# Supplementary material for: Incidence, Risk Factors and Prediction of Secondary Hyperparathyroidism in Preterm Neonates under 32 Weeks’ Gestational Age
Source: Nutrients. 2022 Aug 18;14(16):3397. doi: 10.3390/nu14163397 (PMC9412605; doi:10.3390/nu14163397)
Supplement: Supplementary file 1 [file nutrients-14-03397-s001.zip › nutrients-1848346-supplementary.pdf]

Supplementary Table S1. Univariate analysis in relation to HPTH.

**Perinatal characteristics**	**HPTH *(n=62)***	**NO HPTH *(n=92)***	**p-value**
**X̅± SD**			
Gestational age	29.02 ± 1.90	30.16 ± 2.07	<0.001
Birth weight	1089.35 ± 259.58	1217.01 ± 248.22	0.630
Weight Z-score at birth	-0.39 ± 0.89	-0.56 ± 0.66	0.230
Length Z score at birth	0.03 ± 1.33	0.07 ± 1.06	0.474
Z score at birth	-0.31 ± 1.06	-0.41 ± 1.15	0.564
Maternal age (years)	32.45 ± 6.73	34.14 ± 6.00	0.148
Maternal BMI	27.47 ± 7.61	24.73 ± 4.67	0.030
**No. (%)**			
Maternal hypertension	17 (27.4)	17 (0.18)	0.190
Chorioamnionitis	10 (16.1)	11 (12.0)	0.459
Maternal smoking	7 (11.3)	11 (12.0)	0.842
Vaginal delivery	11 (17.7)	30 (32.6)	0.041
Sex (females)	33 (53.2)	48 (52.2)	0.285
IVF	13 (21.0)	24 (26.1)	0.466
IUGR	9 (14.5)	14 (15.2)	0.905
Moderate-severe IUGR	4 (6.5)	6 (6.5)	0.986

Antenatal steroids	60 (96.8)	90 (97.8)	0.687
Oxygen during resuscitation	58 (93.5)	70 (76.1)	0.005
Intubation in delivery room	15 (24.2)	9 (9.8)	0.016
**Neonatal evolution**	**HPTH *(n=62)***	**NO HPTH *(n=92)***	**p-value**
**X̄± SD**			
IMV hours	142.40 ± 177.44	209.50 ± 277.46	0.786
NIMV hours	247.82 ± 218.86	164.69 ± 163.47	0.023
Hours of oxygen therapy	700.27 ± 688.59	514.07 ± 618.49	0.190
Maximum FiO_2	37.71 ± 13.55	34.17 ± 12.52	0.095
Z-score weight at discharge	-1.37 ± 1.31	-1.50 ± 1.07	0.674
Admission (days)	71.80± 95.254	51.26 ± 20.99	0.003
**No. (%)**			
IMV	35 (56.5)	20 (21.7)	<0.001
NIMV	42 (67.7)	37 (40.2)	0.001
Oxygen therapy	51 (82.3)	60 (65.2)	0.021
Surfactant	38 (61.3)	32 (34.8)	0.001
BPD	24 (38.7)	16 (17.4)	0.003
Corticosteroids for BPD	10 (16.1)	8 (8.7)	0.159
PDA	15 (24.2)	10 (10.9)	0.028

PDA ligation	8 (12.9)	3 (3.3)	0.023
NEC	6 (9.7)	2 (2.2)	0.040
Anemia requiring transfusion	50 (80.6)	50 (54.3)	0.001
Nosocomial sepsis	31 (50.0)	26 (28.3)	0.006
ROP ≥II	12 (19.5)	5 (5.4)	0.040
PVL	3 (4.8)	2 (2.2)	0.360
Home oxygen	6 (9.7)	1 (1.1)	0.012
Furosemide	1 (1.6)	5 (5.4)	0.229
Thiazides	3 (4.8)	2 (2.2)	0.360
Spironolactone	1 (1.6)	3 (3.3)	0.528
Breastfeeding fortification	44 (71.0)	80 (87.0)	0.014
Oral P supplements	20 (32.3)	31 (33.7)	0.853
Oral Ca supplements	50 (80.6)	80 (87.0)	0.290
Oral supplements Vit D	60 (96.8)	92 (100.0)	0.083
MBD	7 (11.3)	15 (16.3)	0.383
**Biochemical and screening parameters**	**HPTH (n=62)**	**NO HPTH *(n=92)***	**p-value**
$\bar{X}$± SD			
Serum P (mg/dl)	6.37 ± 1.04	5.78 ± 1.22	0.013
Serum Ca (mg/dl)	9.65 ± 0.67	9.88 ± 0.50	0.043

Vitamin D (mg/ml)	32.14 ± 11.59	36.24 ± 11.95	0.044
ALP (IU/L)	815.5 ± 326	890.9 ± 385	0.195
Urine P (mg/dl)	14.92 ± 14.52	9.52 ± 12.11	0.029
Urine Ca (mg/dl)	5.68 ± 4.44	8.91 ± 6.58	0.002
Ca/Cr urine	0.55 ± 0.32	1.22 ± 1.96	0.002
P/Ca urine	3.55 ± 5.01	1.62 ± 2.94	< 0.001

BMI = Body Mass Index; IVF = In-vitro fertilization; IUGR =Intrauterine growth restriction; IMV = Invasive mechanical ventilation; NIMV = Non-invasive mechanical ventilation; NICU = Neonatal intensive care unit; MBD = Metabolic bone disease; BPD = Bronchopulmonary dysplasia; PDA = Patent ductus arteriosus; NEC = Necrotizing Enterocolitis; ROP = Retinopathy of Prematurity; IVH = Intraventricular hemorrhage; PVL =Periventricular Leukomalacia; ALP = Alkaline phosphatase; PTH = Parathyroid hormone; Ca = Calcium; P = Phosphorus; Cr = Creatinine
